# Polarized Raman scattering study of kesterite type Cu_2_ZnSnS_4_ single crystals

**DOI:** 10.1038/srep19414

**Published:** 2016-01-18

**Authors:** Maxim Guc, Sergiu Levcenko, Ivan V. Bodnar, Victor Izquierdo-Roca, Xavier Fontane, Larisa V. Volkova, Ernest Arushanov, Alejandro Pérez-Rodríguez

**Affiliations:** 1 Institute of Applied Physics, Academy of Sciences of Moldova, Chisinau, MD 2028, Moldova; 2 Helmholtz Zentrum Berlin fur Materialien und Energie, Berlin, D-14109, Germany; 3 Department of Chemistry, Belarusian State University of Informatics and Radioelectronics, Minsk, Belarus; 4 IREC, Catalonia Institute for Energy Research, Sant Adrià del Besòs (Barcelona), 08930, Spain; 5 IN2UB, Departament d’Electrònica, Universitat de Barcelona, Barcelona, 08028, Spain

## Abstract

A non-destructive Raman spectroscopy has been widely used as a complimentary method to X-ray diffraction characterization of Cu_2_ZnSnS_4_ (CZTS) thin films, yet our knowledge of the Raman active fundamental modes in this material is far from complete. Focusing on polarized Raman spectroscopy provides important information about the relationship between Raman modes and CZTS crystal structure. In this framework the zone–center optical phonons of CZTS, which is most usually examined in active layers of the CZTS based solar cells, are studied by polarized resonant and non-resonant Raman spectroscopy in the range from 60 to 500 cm^−1^ on an oriented single crystal. The phonon mode symmetry of 20 modes from the 27 possible vibrational modes of the kesterite structure is experimentally determined. From in-plane angular dependences of the phonon modes intensities Raman tensor elements are also derived. Whereas a strong intensity enhancement of the polar E and B symmetry modes is induced under resonance conditions, no mode intensity dependence on the incident and scattered light polarization configurations was found in these conditions. Finally, Lyddane-Sachs-Teller relations are applied to estimate the ratios of the static to high-frequency optic dielectric constants parallel and perpendicular to ***c***-optical axis.

The Cu_2_ZnSnS_4_ (CZTS) quaternary compound is widely discussed over the last few years as an active layer in thin films photovoltaic devices^1^,^2^,^3^,^4^,^5^,^6^,^7^,^8^,^9^,^10^,^11^. Although the electronic properties of the CZTS-heterojunction solar cells have been studied extensively the knowledge of the CZTS basic properties is still limited. Particularly, the crystal structure^4^,^12^, intrinsic optoelectronic^5^,^6^,^8^,^9^,^13^,^14^,^15^ and electrical properties^9^,^10^,^16^,^17^ are not well understood. The development of photovoltaic technologies based on this semiconductor strongly requires for the availability of experimental techniques suitable for the analysis of the crystalline quality of the CZTS absorbers. Recently, Raman spectroscopy (which is a nondestructive optical technique) has showed a high potential to be used for characterization of the structure disorder^18^,^19^,^20^, secondary phases^21^,^22^, stress, compositional effects and phonon confinement effects in CZTS semiconductor^23^.

In 1991 Himmrich and Haeuseler first reported characteristic Raman peaks at 285, 336 and 362  cm^−1^ for CZTS powdered samples^24^. Since then several of papers with CZTS lattice vibration spectra have been published, but in most of these reports Raman spectroscopy serves for samples characterization and establishing link between process parameters and films growth^21^,^22^. A more quantitative Raman analysis can be found only in few reports^23^,^25^, which include a very detailed analysis of the vibrational properties of CZTS from the simultaneous fitting of the Raman spectra performed with six different excitation wavelengths on device grade polycrystalline layers^25^. In this work, a first assignment of the symmetry of the modes is made, based in the comparison with theoretical calculations reported in the literature and with polarization measurements, and the presence of phonon confinement effects characteristic of nano-crystalline layers or related to the presence of structural defects has also been analysed in^23^. As reported in this work, these effects determine a broadening and a shift to lower frequencies of the main Raman peaks, and take place for samples with crystal sizes lower than 50 nm.

However, polarization measurements performed on polycrystalline layers have limitations for the assignment of the symmetry of Raman peaks, because of the randomly oriented grains in the layers. Unambiguous determination of the symmetry and selection rules requires the use of single crystal samples with well-known crystalline orientation. Recent Raman scattering experiments on CZTS single crystals have provided clear evidence for a strong polarization dependence of the Raman peaks on the crystallographic directions of the investigated crystal^26^. However, the valuable information on the Raman tensor elements is not given and phonon modes below 140  cm^−1^ are not studied in this publication.

Here we present the polarized Raman measurements under non-resonant and resonant conditions on oriented CZTS single crystal in the range of 60–500  cm^−1^. The analysis of Raman mode intensities as a function of in plane angle of the (1 1 2)-plane allows us to determine the symmetry of the phonon modes. As a result the identification of 20 from the 27 possible modes of the kesterite structure is performed, completing the previous identification that was reported in^25^,^26^, and the ratios of Raman tensor elements are evaluated for many of them.

## Results and Discussions

The CZTS compound crystallizes in the kesterite type structure, space group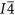. For this space group, taking into account the eight atoms site position in the CZTS unit cell, the group theory^27^ yields the following irreducible representation of the twenty four phonons at the center of the Brillouin zone, Г point (see Supplementary information)


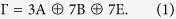


**Figure 1:**
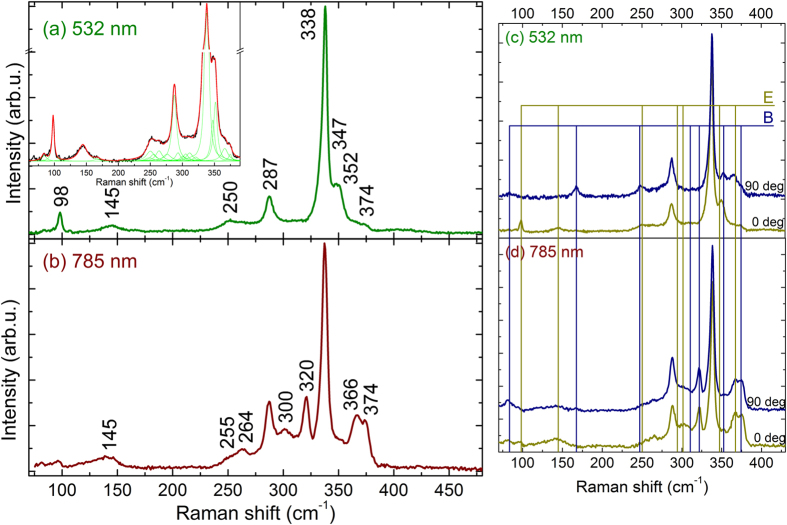
Raman spectra of Cu_2_ZnSnS_4_ single crystals excited with laser line 532 nm (a) and 785 nm (b), close to resonant conditions. Inset of figure (**a**) shows the fitting (red curve) of the experimental Raman spectra (black curve) with Lorentzian curves (green). Here the breaks on intensity scales were performed to show weaker peaks with lower intensity. Figures (**c**,**d**) represent the spectra measured at different polarization configurations. Here angles 0 deg and 90 deg correspond to 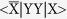 and 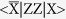 configurations, respectively.

Here 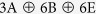 modes are Raman active, from them 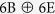 modes are also IR-active, which leads to their LO-TO splitting. E modes are also double degenerated. The rest 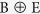 modes are acoustic. The intensity of a Raman mode depends on the polarizations of the incident and scattered light and the Raman tensor elements for the crystal plane on which measurements are performed (see Supplementary information). It is also possible to distinguish between the B and E symmetry modes by carrying out backscattering measurements on (1 1 2) crystal facet as it has been recently shown for the close related kesterite Cu_2_ZnSnSe_4_ (CZTSe)^28^. The non-polarized Raman spectrum of CZTS single crystal is presented in Fig. 1 (a). Most intense peaks are in accordance with data from previously reported studies on single crystals^26^, thin films^21^,^22^,^25^ and powder samples^18^,^24^,^29^. It should be noted, that the number of the experimental modes in the region 220–330 cm^−1^ are less than the theoretically predicted: two A modes, two polar B (TO/LO) modes and two polar E (TO/LO) modes for the kesterite type structure^30^,^31^,^32^. The small Raman cross section of the missing Raman lines and possible overlapping in the position of the peaks might be reasons, why these modes are not clearly seen in the spectra. To resolve this issue we have made resonance Raman scattering measurements using excitation photons with energy close to the band gap transition in CZTS. In these conditions, an enhancement in the intensity of some of the polar modes is expected^33^,^34^,^35^,^36^. The non-polarized resonant Raman spectrum of CZTS single crystal is shown in Fig. 1(b). New peaks at 255, 264, 300, 320 and 366 cm^−1^ are clearly identified in the spectrum. In addition, there is also a strong increase in the intensity of the peak at 374 cm^−1^. We assume that these peaks are LO components of the polar B and E symmetry modes, which gain their intensity due to Fröhlich electron-phonon interaction^33^. Raman spectra were fitted with Lorentzian functions (an example of such decomposition is presented in the inset of Fig. 1 (a)) based on the phonon modes positions found under resonance and non-resonance conditions. The full width at the half maximum (FWHM) of the most intense peaks was in the range 5–8 cm^−1^, which confirms the good crystallinity of the grown samples. In the 230–300 cm^−1^ spectral region, some phonon modes have larger FWHM values (10–15 cm^−1^), which might be due to the presence of the additional multiphonon peaks^37^. It is interesting to remark that weaker peaks at 643 and 740 cm^−1^ (not shown here) have also been detected. These peaks match well with the second order of the resonant enhanced modes at 320 and 374 cm^−1^, respectively. Recently high overtones phonon modes were also found for CZTS crystals at 664, 968 and 1323 cm^−1^ 38.

The Raman spectra measured with 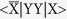 and 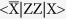 polarization configurations, according to Porto notations^39^, are plotted in Fig. 1(c,d). The two most intense peaks at 287 and 338 cm^−1^ are clearly identified with the dominant non-polar A-symmetry modes. Based on the selection rules (see Supplementary Table S3) for non-resonance condition Raman peaks at 84, 167, 248 and 352 cm^−1^ visible in the 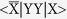 spectra are attributed to B-symmetry modes, while those peaks at 83, 98, 145 and 347 cm^−1^ visible in the 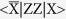 spectra are identified with E-symmetry modes. However, under resonant excitation conditions all Raman peaks maintain near the same intensity in both 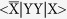 and 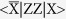 configurations (Fig. 1(d)). These data show a violation under resonant conditions of the selection rules for the polar B and E symmetry modes. For close related Cu(In,Ga)S_2_ chalcogenides it was found similarity in selection rules for resonant Raman scattering and optical transitions near band edge^40^,^41^. Thus we may assume that the absence of the polarization behavior in CZTS resonant Raman spectra is due to a small anisotropy of the optical transitions relative to the crystal axis ***c*** as the incoming photon with **Y** polarization is perpendicular to ***c***, while the incoming photon with **Z** polarization has an angle of 35° with the ***c*** axis. By decomposition of Raman spectra with the Lorentzian curves the phonon frequencies of the different modes are obtained and are summarized in Table 1. Here the theoretical results published in ref. 32 are also given and the symmetry of modes indicated in italic is proposed based on this work. It should be noted that position of LO modes was determined from the resonant Raman (RR) spectra, while TO modes were identified based on theoretical calculations^30^,^31^,^32^. As shown, the TO-LO splitting does not exceed 16 cm^−1^ even for the modes with highest wavenumber. Thus in the fitting of the spectra we added the peaks of the TO components close to the LO ones until the quality of the fitting was not improved. However, the estimated position of the TO components can have an uncertainty in the range of 1–2 cm^−1^.

The identification of the exact position of a third A symmetry mode in the region 220–330 cm^−1^ was more complex from the analysis of our measurements. Dimitrievska *et al.*^25^ identified this mode with a peak located at 306 cm^−1^ that appears mainly when measuring with 633 nm and 785 nm excitation wavelengths, and this identification was proposed mainly from comparison with the theoretical data reported in ref. 30. However, the analysis of the spectra measured on the single crystal samples has not allowed to corroborate this identification. On the other hand, the detailed fitting of the experimental polarization spectra measured with different angles on the (1 1 2) crystal plane suggests the presence of a contribution at 276 cm^−1^ with A symmetry. The assignment of this contribution with the third A symmetry mode of the kesterite structure agrees with the fact that the theoretical calculations in ref. 32 predict two A modes relatively close (at 270.0 cm^−1^ and 281.7 cm^-1^) and a mode that is more separated at higher frequencies (at 338.5 cm^−1^, corresponding to the dominant mode in the spectra). Similar results were predicted from other theoretical calculations^31^. This also agrees with the experimental identification in the close related CZTSe kesterite, where two close spaced A-modes at lower frequencies were found^28^.

**Figure 2:**
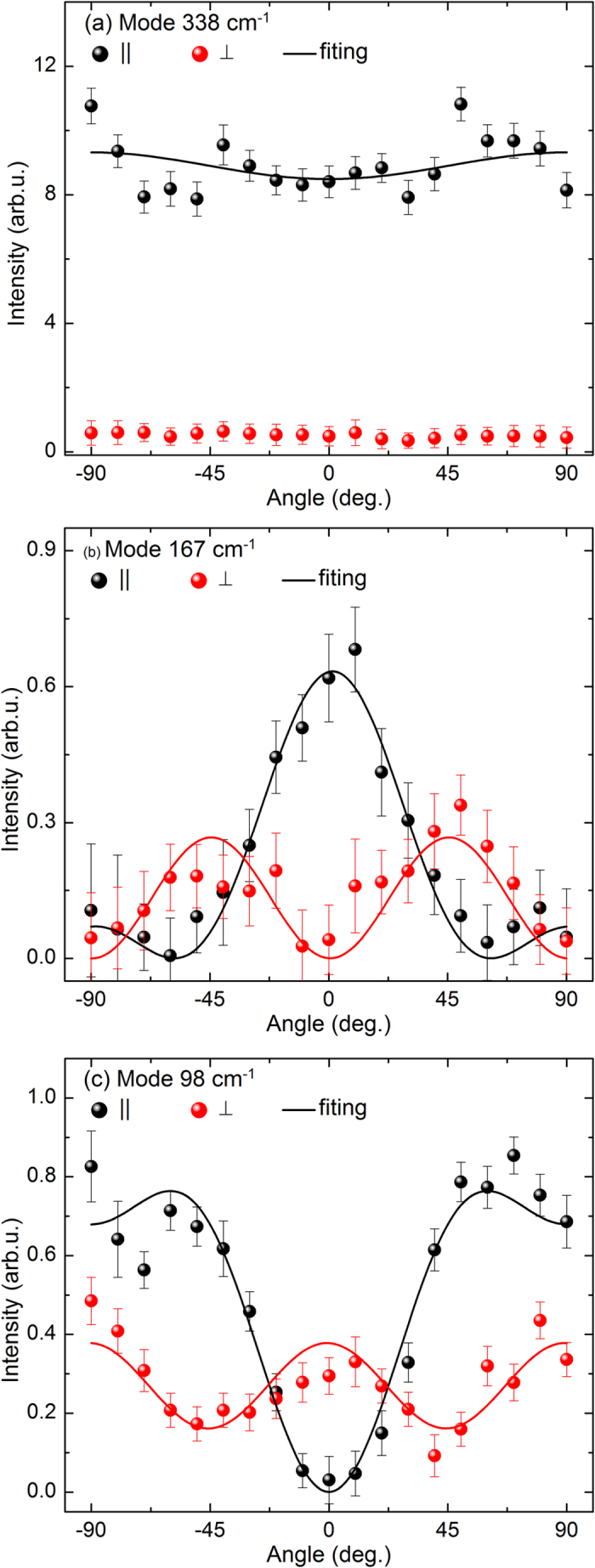
Examples of the angular dependence of the Raman peaks intensities of the A (a), B (**b**) and E (**c**) mode symmetry. Solid lines are fitting to corresponding Eqs. from Supplementary Table S3 for parallel and perpendicular configurations.

The accurate analysis of the polarized Raman spectra measured at different angles *θ* on the (1 1 2) plane has allowed to corroborate the symmetry assignment of several of the vibrational modes. Fitting of the intensity of the peaks as a function of the in plane angle *θ*, following the equations derived in ref. 28 (see the Supplementary Table S3) for the single crystal CZTSe kesterite with the similar crystalline orientation has allowed also to make an estimation of the ratios of the Raman tensor elements as indicated in Table 1. Examples of angular dependencies obtained for modes with different symmetry are exhibited in Fig. 2, where the solid lines correspond to the fitting of the experimental data with the equations derived in ref. 28. Here we see that in case of the A symmetry modes their intensity is almost independent on the rotation angle in parallel configuration and is close to zero in perpendicular configuration (Fig. 2(a)). Similar results are found in case of other kesterite type compounds as CZTSe and Cu_2_ZnGeSe_4_^28^ and can be explained by the close values of *a* and *b* tensor elements, i.e. *a*/*b* ≈ 1. The fitting of angular dependence of some B and E symmetry modes was also performed (see examples at Fig. 2 (b,c)). For these modes the obtained results are also similar to those previously reported for the kesterite CZTSe and Cu_2_ZnGeSe_4_ compounds^28^, with a strong anisotropy for the tensor elements of B symmetry modes and a much smaller anisotropy for tensor elements of E symmetry modes. This appears as a characteristic property of kesterite type quaternary compounds. Since the properties of the phonons are directly related with Raman tensor elements, the estimated ratio of the tensor elements could be useful for the prediction of the mode intensities or angular intensity patterns for others crystal geometries. For instance, Raman measurements on (0 0 1)-plane with parallel geometry (incident and scattered light polarization either along [1 0 0] or [0 1 0]) should result in very low B-mode intensities in comparison to the cross geometry-case due to large anisotropy for B-mode tensor elements. Indeed the latter B-mode behavior was observed in polarization resolved experiment in ref. 26, while no explanation was given.

Finally, the analysis of the frequencies of the polar modes has allowed us to estimate the ratio of the static (ε_0_) to optic (ε_∞_) dielectric constants. This is based on the Lyddane-Sachs-Teller relations for uniaxial crystals as^42^:


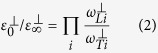



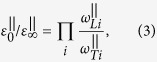


where *i* denotes polar mode, 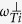
(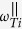) and 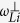
(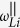) are the frequencies of TO and LO components of the E (B)-symmetry modes, respectively. As it seen from the Table 1 we resolved most of E (B) TO/LO peaks and substituting these values to Eqs. (2) and (3) we found experimental ratios of 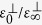 ≈ 1.14 and 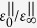 ≈ 1.12. These results are in good agreement with the values 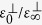 ≈ 1.12 and 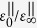 ≈ 1.14 obtained from the substitution of theoretical phonon frequencies (Table 1, ref. 32). Theoretical values for static dielectric constant 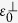 and 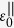 are 9.1 and 9.8^4^ and high frequency dielectric constants 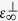 and 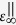 are 6.8 and 6.6^43^, which results in larger ratios 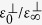 ≈ 1.34 and 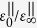 ≈1.48 than those determined from phonon frequencies. This found discrepancy is most likely related to some overestimation in calculation of the 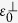 and 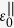 constants performed in ref. 4, while 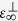 and 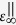 values^31^ agree well with 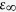 ≈ 7 measured by ellipsometry on CZTS^44^.

## Conclusions

Summarizing results presented above, from the polarized Raman scattering investigations performed on Cu_2_ZnSnS_4_ single crystals we experimentally determined the symmetry of 20 from 27 possible modes. The third A symmetry mode frequency was found at 276 cm^−1^, which was supported by previously published *ab-initio* calculations. The ratio of Raman tensor elements were obtained for most of the observed peaks and a strong difference in *c* and *d* elements of polar B modes and no pronounced difference of *a* and *b* and of *e* and *f* elements of non-polar A and polar E modes was revealed. For the resonant conditions Raman scattering measurements revealed an enhancement in intensity of the polar E and B modes, which under these conditions do not follow the selection rules. In addition, the ratios of the static to high-frequency optic dielectric constants parallel and perpendicular to the ***c***-crystallographic direction were also extracted by using Lyddane-Sachs-Teller relations. The Raman scattering results obtained in the present study could be very useful for the determination of the crystallographic orientations in CZTS semiconductor in bulk and thin film forms.

### Experimental details

**Figure 3:**
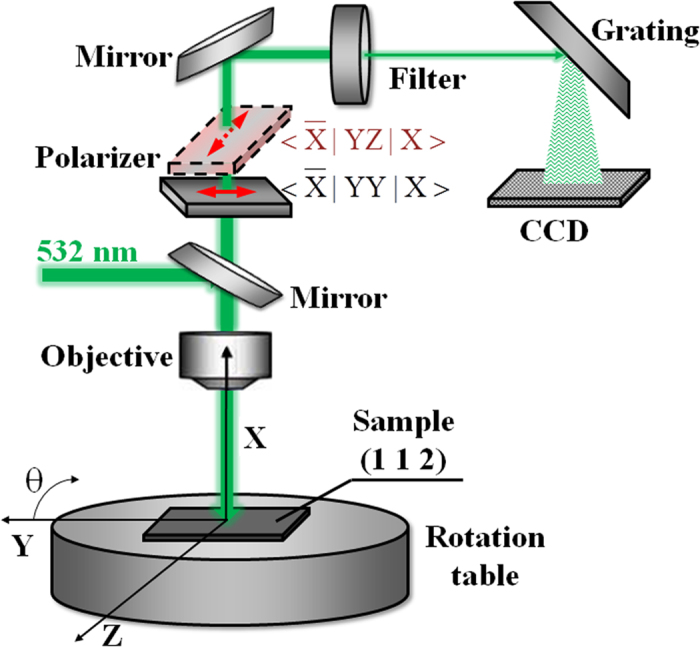
Schematic representation of the experimental configuration used in the polarized Raman experiments performed on the (1 1 2) plane.

The CZTS single crystals were grown by chemical vapor transport using iodine as a transporter. The pure elements of Cu, Zn, Sn and S in the stoichiometric proportion were placed in evacuated ampoule together with 5 mg/cm^3^ of iodine. The growth process was performed in the vertical two zone furnace during 14 days, and the evaporation and growth temperature were ~800 °C and ~750 °C, respectively. The chemical composition of the bulk single crystals was determined by using X-ray fluorescence. The selected samples were close to stoichiometry (Cu:Zn:Sn:S = 25.3:13.0:11.7:50.0, all values in at.%). The crystal orientation was determined by rotating orientation XRD method, and it revealed that the crystal basal plane is (1 1 2) and the long edge of the crystal platelet is along 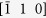 direction. All Raman spectra were taken in the backscattering configuration and (**X Y Z**) laboratory coordinate system were used (see Fig. 3). Here **X, Y** and **Z** lie along the 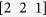, 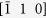 and 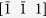 crystallographic directions.

Raman spectra were excited by a YAG:Nd solid state laser (line 532 nm) and recorded using a LabRam HR800-UV Horiba Jobin Yvon spectrometer in conjunction with a CCD detector. For the resonant Raman (RR) spectra a solid state laser (line 785 nm) as an excitation source was used. All spectra were measured in backscattering configuration through an Olympus metallographic microscope with convergence power ×50. By using 1800 groove/mm grating and a narrow slit (150 μm), a spectral resolution of ~1 cm^−1^ was achieved. In the measurements the laser power was ~1 mW. At these excitation conditions no thermal effects are observed on the sample surface. Figure 3 shows a schematic representation of the experimental configuration used in these measurements. Note, that polarization of the incident laser beam was fixed and aligned along **Y** axis. The polarization of the scattered beam was changed by using a parallel (gray) and a cross (reddish) polarizer, which were used for the parallel and perpendicular configurations, respectively. In terms of Porto notations^39^ the light propagation in parallel configuration corresponds to the 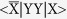 case, while the cross geometry corresponds to the 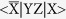 case (Fig. 3). Here the letters outside the line brackets indicate the direction of the incident and scattered light, respectively, while letters inside the line brackets indicate the polarization of the incident and scattered light, respectively^39^.

## Additional Information

**How to cite this article**: Guc, M. *et al.* Polarized Raman scattering study of kesterite type Cu_2_ZnSnS_4_ single crystals. *Sci. Rep.* **6**, 19414; doi: 10.1038/srep19414 (2016).

## Supplementary Material

Supplementary Information

## Figures and Tables

**Table 1:** Symmetry of the Raman peaks of Cu_2_ZnSnS_4_ kesterite type quaternary compound and ratios of Raman tensor elements.

A		*a/b*||/⊥	B (TO+LO)		*c/d*||/⊥	E (TO+LO)		*e/f*||/⊥
Theor	Exper		Theor	Exper		Theor	Exper	
270.0	276	—	87.8 + 88.2	**84**	<0.1/0.13 ± 0.09	82.2 + 82.2	**83**	0.97 ± 0.09/0.85 ± 0.15
281.7	**287**	1.28 ± 0.07/−	99.3 + 99.3	—	—	102.9 + 103.0	**98**	0.92 ± 0.03/1.10 ± 0.05
338.5	**338**	0.93 ± 0.04/−	168.2 + 169.5	**167**	<0.1	150.0 + 150.5	**145**	0.92 ± 0.06/1.12 ± 0.06
			237.9 + 253.7	**248** + 255	<0.1	247.8 + 254.8	250/*264*	—
			307.6 + 311.4	*311* + *320*	—	278.0 + 290.3	*293/300*	—
			357.0 + 373.6	**352** + *374*	<0.1	351.1 + 365.3	**347**/*366*	0.89 ± 0.05/0.85 ± 0.07

Peaks position indicated in italic correspond to peaks where a tentative symmetry assignment is proposed based on comparison with theoretical calculations^32^. For peaks position indicated in bold the ratio of Raman tensor elements was calculated.
