# Supplementary material for: Polarized Raman scattering study of kesterite type Cu2ZnSnS4 single crystals
Source: Sci Rep. 2016 Jan 18;6:19414. doi: 10.1038/srep19414 (PMC4726006; doi:10.1038/srep19414)
Supplement: Supplementary Information [file srep19414-s1.doc]

**Polarized Raman scattering study of kesterite type Cu_2_ZnSnS_4_ single crystals**

**Maxim Guc^1,4,*^, Sergiu Levcenko^2^, Ivan V. Bodnar^3^, Victor Izquierdo-Roca^4^, Xavier Fontane^4^, Larisa V. Volkova^3^, Ernest Arushanov^1^, Alejandro Pérez-Rodríguez^4,5^**

**Supplementary information**

The group theoretical analysis [27] for the zone center phonons, applied to kesterite CZTS (Fig. S1), with respect to Wyckoff position of all atoms (Ref. [14]), are given in Table S1.

**Table S1.** Atomic coordinates, Wyckoff Position, atom sites symmetry and irreducible representations for the atoms of the tetragonal CZTS.

| Atom | Wyckoff position | | Symmetry | Irreducible representations | |
| --- | --- | --- | --- | --- | --- |
| Cu(1) | 2*a* | | $\overline{4}$ | $\text{B}\oplus\text{ E}$ | |
| Cu(2) | 2*c* | | $\overline{4}$ | $\text{B}\oplus\text{ E}$ | |
| Sn(1) | *2b* | | $\overline{4}$ | $\text{B}\oplus\text{ E}$ | |
| Zn(1) | 2*d* | | $\overline{4}$ | $\text{B}\oplus\text{ E}$ | |
| S(1) | *8g* | | $1$ | $3\text{A}\oplus\text{ }3\text{B }\oplus\text{ 3E}$ | |
| Modes classifications | | | | | |
| IR | | Raman | | | Acoustic |
| $\text{6B}\oplus\text{ 6E}$ | | $\text{3A }\oplus\text{ }6\text{B }\oplus\text{ 6E}$ | | | $\text{B }\oplus\text{ E}$ |

**
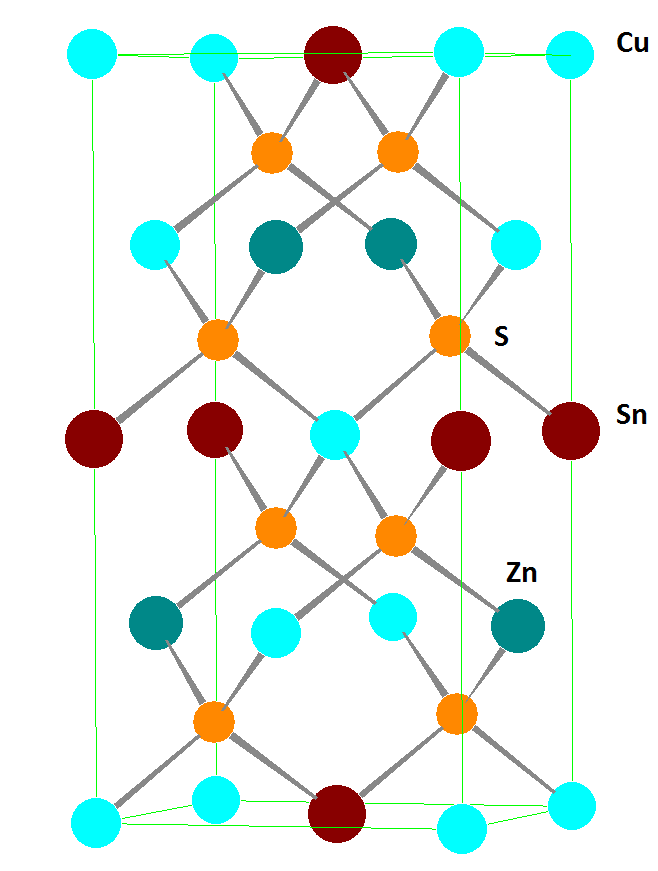
**

**Figure S1.** CZTS kesterite unit cell structure.

The Raman intensity, *I*, is given by [45]

$I\propto\left|\nu_{i}\Re_{XYZ}\nu_{s}\right|^{2},$ (S1)
where *v_i_* is the incident light polarization, *v_s_* is the scattered light polarization and $\Re_{XYZ}$ is the Raman tensor for the phonon mode. Here (X Y Z) is the laboratory system associated with (1 1 2)-crystal plane, where **X, Y** and **Z** correspond to $[2 2 1]$, $[\overline{1} 1 0]$ and $[\overline{1} \overline{1} 1]$ crystallographic directions, respectively. For the arbitrary value of the in-plane angle, θ, the incident and scattered light polarization vectors are defined by

$v_{i}=\left(\begin{matrix} 0 & \cos\theta& \sin\theta\end{matrix}\right)$ $v_{s}^{||}=\left(\begin{matrix} 0 \\ \cos\theta\\ \sin\theta\end{matrix}\right)$ $v_{s}^{\perp}=\left(\begin{matrix} 0 \\ -\sin\theta\\ \cos\theta\end{matrix}\right)$, (S2)

where $v_{s}^{||}$ is parallel and $v_{s}^{\perp}$is perpendicular geometry.

To apply Eq. (S1) we used results of the Ref. [28], where $\Re_{XYZ}$tensors for the kesterite (1 1 2)-plane were already determined. For clarity we provide these $\Re_{XYZ}$ in Table S2, too. Finally, the calculated angular dependence for the **A**, **B** and **E**-symmetry modes and the selection rules for the considered geometries $<\overline{\text{X}}\text{|YY|X}>$ and $<\overline{\text{X}}\text{|ZZ|X}>$are collected in Table S3.

**Table S2.** Calculated Raman tensors for kesterite type structures in case of (1 1 2) crystal plane.

| Mode | Raman tensor |
| --- | --- |
| A | $\left(\begin{matrix} \frac{2a+b}{3} & 0 & \frac{\sqrt{2}}{3}\left(b-a\right) \\ 0 & a & 0 \\ \frac{\sqrt{2}}{3}\left(b-a\right) & 0 & \frac{a+2b}{3} \end{matrix}\right)$ |
| B(Z) | $\left(\begin{matrix} -\frac{2d}{3} & \sqrt{\frac{2}{3}}c & \frac{\sqrt{2}d}{3} \\ \sqrt{\frac{2}{3}}c & d & -\frac{c}{\sqrt{3}} \\ \frac{\sqrt{2}d}{3} & -\frac{c}{\sqrt{3}} & -\frac{d}{3} \end{matrix}\right)$ |
|
| E(X) | $\left(\begin{matrix} \frac{2}{3}(e-f) & \frac{e+f}{\sqrt{6}} & \frac{e-f}{3\sqrt{2}} \\ \frac{e+f}{\sqrt{6}} & 0 & \frac{e+f}{\sqrt{3}} \\ \frac{e-f}{3\sqrt{2}} & \frac{e+f}{\sqrt{3}} & -\frac{2}{3}(e-f) \end{matrix}\right)$ |
| E(Y) | $\left(\begin{matrix} \frac{2}{3}(e+f) & -\frac{e-f}{\sqrt{6}} & \frac{e+f}{3\sqrt{2}} \\ -\frac{e-f}{\sqrt{6}} & 0 & -\frac{e-f}{\sqrt{3}} \\ \frac{e+f}{3\sqrt{2}} & -\frac{e-f}{\sqrt{3}} & -\frac{2}{3}(e+f) \end{matrix}\right)$ |

**Table S3.** Angular dependence of Raman mode intensities for kesterite type structure in case of (1 1 2) crystal plane (upper part of the Table) and intensity values for the selected geometries (lower part of the Table).

| Mode | || | | ⊥ | |
| --- | --- | --- | --- | --- |
| A | $\left\vert\frac{1}{3}\left[2a+b+(a-b)\cos(2\theta)\right]\right\vert^{2}$ | | $\left\vert-\frac{1}{3}(a-b)\sin(2\theta)\right\vert^{2}$ | |
| B(Z) | $\left\vert\frac{1}{3}\left[d+2d\cos(2\theta)+\sqrt{3}c\sin(2\theta)\right]\right\vert^{2}$ | | $\left\vert\frac{1}{3}\left[\sqrt{3}c\cos(2\theta)-2d\sin(2\theta)\right]\right\vert^{2}$ | |
| E(X) | $\left\vert\frac{2}{3}\sin(\theta)\left[\sqrt{3}(e+f)\cos(\theta)+(e-f)\sin(\theta)\right]\right\vert^{2}$ | | $\left\vert\frac{1}{3}\left[\sqrt{3}(e+f)\cos(2\theta)+(e-f)\sin(2\theta)\right]\right\vert^{2}$ | |
| E(Y) | $\left\vert\frac{2}{3}\sin(\theta)\left[\sqrt{3}(f-e)\cos(\theta)+(e+f)\sin(\theta)\right]\right\vert^{2}$ | | $\left\vert\frac{1}{3}\left[\sqrt{3}(f-e)\cos(2\theta)+(e+f)\sin(2\theta)\right]\right\vert^{2}$ | |
| Mode | |  | |  |
| A | | a^2^ | | ((a+2b)/3)^2^ |
| B(Z) | | d^2^ | | d^2^/9 |
| 0.5×E(X)+0.5×E(Y) | | 0 | | (4/9)(f^2^+e^2^) |
